# Influence of Opening Time Interval of Gate Signals on Suppression of Horizontally Polarized Signal of Infrared Pulsed Laser

**DOI:** 10.3390/ma17215276

**Published:** 2024-10-30

**Authors:** Xinyang Wu, Dongdong Wang, Di Song, Jiaqi Wang, Jiawei Guo, Peng Ren, He Cai, Yaqing Jin, Yonghong Yu, You Wang, Jing Liu

**Affiliations:** 1 School of Physical Science and Technology, Xinjiang University, Urumchi 830017, China; wxy@stu.xju.edu.cn; 2 School of Materials Science and Engineering, Xinjiang University, Urumchi 830017, China; 3 Southwest Institute of Technical Physics, Chengdu 610041, China; mingboor@163.com (D.W.); disong2022@alu.scu.edu.cn (D.S.); 15100491399@163.com (J.W.); lcl-330@163.com (J.G.); 18982137452@163.com (P.R.); caihe953@sina.com (H.C.); jinyaqing18@mails.ucas.ac.cn (Y.J.); yonghong-yu@alu.ruc.edu.cn (Y.Y.)

**Keywords:** Nd:YVO_4_ pulsed lasers, “match-head” effect, giant pulse suppression, abnormal horizontally polarized emission, slope duration of RF signals

## Abstract

Since the beginning of the 21st century, infrared Nd:YVO_4_ pulsed lasers have been widely applied, especially in some actual industrial processes. In the working process of a laser-aided etching device, the “match-head” effect must be effectively controlled by suppressing the first giant pulse for a solid-state Q-switched laser. In the process of optimizing the infrared Nd:YVO_4_ pulsed laser by adjusting the slope parameters of the radio frequency (RF) modulation to suppress the first giant pulse, it has been found that an abnormal horizontally polarized emission with a very short time appears before the formal vertically polarized emission when the gate signal is artificially started. Actually, abnormal horizontally polarized emissions will bring some unexpected machining traces during the production process and even greater dangers. The experimental results show that with the increase in the slope duration of an RF signal, the existence time of abnormal output horizontally polarized light will be shortened, and the horizontal giant pulse and vertical giant pulse are well suppressed. When the slope duration is greater than 0.18 ms, both horizontal and vertical giant pulses will disappear. The horizontally polarized light can be thoroughly suppressed when the slope duration is greater than 13.7 ms. Compared with the method of adding a polarizer to eliminate abnormal output horizontally polarized light, this method does not add elements in the laser, ensuring that the laser volume is relatively small, and does not affect the quality of the normal output laser. The research conclusion is thought to be of great practical significance, especially for processing transparent materials.

## 1. Introduction

In recent years, technology and corresponding industry have been developing rapidly. The near-infrared pulsed laser generated from Nd:YVO_4_ media has been used in the field of laser processing for a long time. Nd:YVO_4_ lasers were first developed in the 1960s, limited by the technical level at that time, and such lasers are mainly used in low-power devices. With the rapid development of laser diode (LD) technology, Nd:YVO_4_ lasers have been gradually turning to LD pumping, forming all-solid-state lasers. This pumping mode has the advantages of high efficiency, good stability, compact structure and long life, which greatly improves the performance of lasers. In recent years, all-solid-state Nd:YVO_4_ lasers have been widely used in micro and nano processing, information communication, biomedicine and scientific research. With the continuous development of technology, higher-power, higher-efficiency and more compact Nd:YVO_4_ lasers have been introduced. In the future, with the continuous emergence of new materials and new technologies, Nd:YVO_4_ lasers will be combined with new technologies and new materials, such as quantum dots and graphene, to further expand the application field of the laser and improve the performance level.

Nd:YVO_4_ lasers show significant advantages and potential in precision wafer cutting technology. In order to improve the cutting accuracy and stability of Nd:YVO_4_ lasers, it is necessary to further improve their output beam quality and thermal management technology. This research aims to solve the problem of the unstable beam quality of Nd:YVO_4_ lasers in the process of precision wafer cutting, restrain the appearance of abnormal machining scars and further improve the machining accuracy of Nd:YVO_4_ lasers. In order to improve the output quality of a 1342 nm Nd:YVO_4_ pulse laser and avoid the unexpected marks produced during the laser processing, it is often necessary to suppress the giant pulse of a Q-switched laser at the beginning of a gate signal working [1]. Actually, the traditional first pulse suppression methods include reducing the peak power of optical pulses by decreasing the pulse width of the modulated signal or changing the pump threshold to make the optical pulse width increase and the peak power decrease [2]. Generally, the conventional methods for the first pulse suppression have high complexity and poor stability. Furthermore, some researchers have found that the polarization status of Nd:YVO_4_ pulse lasers is often very complicated at the beginning of a gate signal working. This means that, except for the first pulse suppression, investigating the polarized directions of Nd:YVO_4_ pulsed lasers is essential during the period when gate signals begin to work.

In this experiment, the authors selected a-cut Nd:YVO_4_ as the laser crystal. According to the results of previous research [3,4], when the pump light is vertically polarized, the laser output generated by the a-cut Nd:YVO_4_ crystal is also vertically polarized light. However, our team has found that, surprisingly, the strange horizontally polarized light can also be outputted before the general radiation of vertical polarized light. This part of the horizontally polarized light is an abnormal output, which will cause unexpected machining scars when the a-cut Nd:YVO_4_ laser is used for the wafer cutting. Therefore, it is necessary to eliminate the abnormal output of horizontally polarized light during the relevant applications. In fact, such horizontally polarized light outputted from an a-cut Nd:YVO_4_ laser was discovered by the authors for the first time, and no relative research has been reported on this issue until now.

In this paper, we focus on studying the relationship between the slope duration of an RF signal of an acousto-optical (AO) Q-switch [5] and the abnormal output horizontally polarized light, as well as the first giant pulses. Through the experiments, we finally found that both the abnormal output horizontally polarized light and the giant pulse can be effectively suppressed by extending the slope duration of an RF signal. In such a scheme, it is unnecessary to increase any optical elements. The optical system can be constructed in a small volume, and the power and other performances of the outputted vertically polarized light will not be affected anymore. This study provides a protocol for guaranteeing the quality and safety of our Nd:YVO_4_ laser for on-line industrial processing. The conclusions in this paper are thought to be valuable for the design of other types of solid-state lasers.

## 2. Theoretical Analyss

An LD end-pumped AO Q-switched laser was used in this experiment. Nd:YVO_4_ is the normal gain medium of a 1342 nm near-infrared laser, which is a kind of tetragonal crystal with a zircon structure, belonging to a uniaxial crystal system. Nd^3+^ in the crystal has low point group symmetry and high ion oscillation intensity. The YVO_4_ substrate sensitizes Nd^3+^ and improves the absorption capacity of activated ions. The crystal has the characteristics of a wide absorption bandwidth, large excited cross-sectional area, large absorption coefficient and polarization output. In addition, the wide absorption bandwidth of Nd:YVO_4_ crystals [6] is easily matched with the pumping wavelength of LDs, which catches the satisfactory pump efficiency.

### 2.1. Kinetic Principles of Nd:YVO_4_

**Figure 1:**
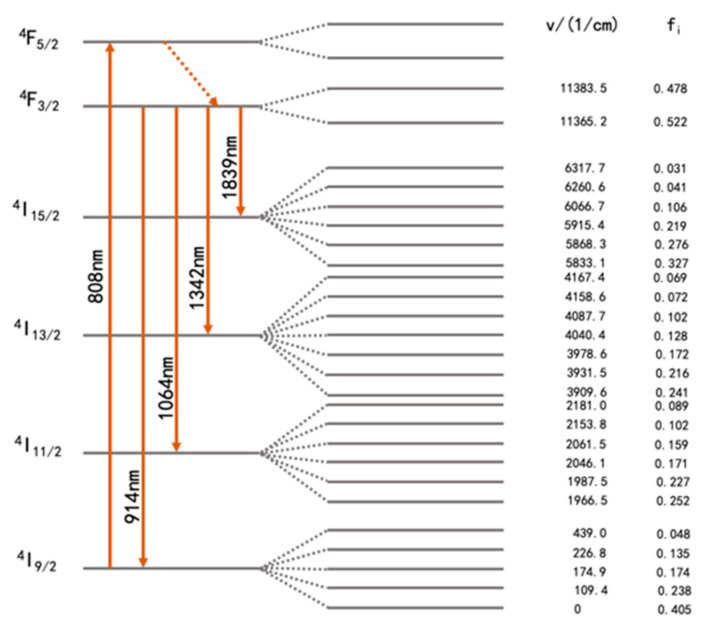
The energy level structure of the Nd:YVO_4_ crystal (v: the energy of each sub-level; f_i_: the proportion of the sub-level in the main level).

The strongest absorption wavelength of Nd:YVO_4_ is located at 808 nm, and the absorption width is about 20 nm. The wavelength of LD emission is adjusted to 808 nm to make it consistent with the strongest absorption peak of the Nd:YVO_4_ crystal. The energy level structure of the Nd:YVO_4_ crystal is shown in Figure 1. By using an 808 nm LD pump source, the electrons from the ground state can be stimulated to the ^4^F_5/2_ level. Since the lifetime of the upper level is very short, the electrons will choose the relaxation process without any radiative emission to reach the metastable ^4^F_3/2_ level. The four emission spectral lines of Nd:YVO_4_ are shown in Figure 1. The infrared laser corresponding to the emission spectral lines of ^4^F_3/2_—>^4^I_13/2_ is selected for obtaining the 1342 nm radiation in this experiment.

**Figure 2:**
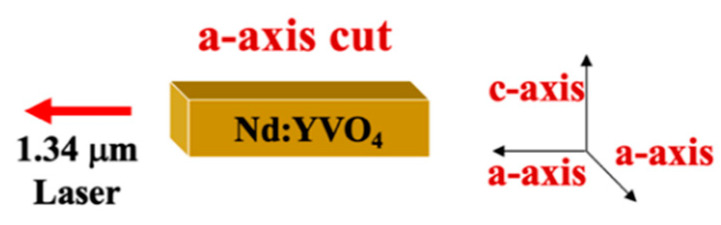
The axial structure of the Nd:YVO_4_ crystal in our study.

The axial structure of the Nd:YVO_4_ crystal in our experiment is shown in Figure 2.

The crystal was cut along the a-axis, and the excited cross-section area of the Nd:YVO_4_ crystal in the direction of the c-axis is about ten times as great as that of the a-axis [7]. Therefore, in the stable pulse oscillation process, the output is a vertically polarized laser at 1342 nm in most cases. However, the giant pulses and abnormal horizontally polarized lasers were also observed before the desired vertical polarized laser output for processing applications due to the particularity of the wavelength of 1342 nm.

### 2.2. Solution of Rate Equations of a Q-Switched Laser

Bragg diffraction explains the main working principle for an AO Q-switch. The laser beam passing through the ultrasonic field in the AO medium is deflected out of the resonant cavity, thus increasing the loss of the resonant cavity. So, the Q value dramatically decreases, and the laser oscillation cannot be formed at that time. After the number of electrons in the upper level of the working substance continuously accumulates to the maximum, if one removes the ultrasonic field in the AO medium as fast as possible, the loss in the cavity instantly decreases and the Q value increases. Laser oscillation can be quickly established, and a 1342 nm laser is therefore outputted [8].

The number of photons in the cavity, Φ, varies with the inversion particle number, N, as expressed by

(1) $$\frac{d\Phi }{dN}=\frac{1}{2}\left(\frac{N_{th}}{N}-1\right)$$

where *N_th_* is the number of electrons at the lasing threshold. By integrating $\frac{d\Phi }{dN}$, we can obtain

(2) $$\Phi -\Phi_{i}=\frac{1}{2}\left(N_{i}-N+N_{th}ln\frac{N}{N_{i}}\right)$$

where $\Phi_{i}$ is the initial photon number in the cavity and $N_{i}$ is the number of electrons initially reversed. As $\Phi_{i}=0$ when the Q value changes by step, then

(3) $$\Phi =\frac{1}{2}\left(N_{i}-N+N_{th}ln\frac{N}{N_{i}}\right)$$

The above formula indicates that the number of photons in the cavity, Φ, varies with the inversion particle number N reserved by the working substance. When the initial inversion particle number N equals the threshold inversion particle number $N_{th}$, the number of photons in the cavity reaches the maximum $\Phi_{m}$ as given by

(4) $$\Phi_{m}=\frac{1}{2}\left(N_{i}-N_{th}+N_{th}ln\frac{N_{th}}{N_{i}}\right)$$

When the number of photons in the cavity reaches the maximum $\Phi_{m}$, the power of the giant pulse output by the output coupler (OC) reaches the maximum value, $P_{m}$, as expressed by

(5) $$P_{m}=\hbar \nu \Phi_{m}\delta_{0}$$

where ℏ is the Planck’s constant, ν  is the laser oscillation frequency and $\delta_{0}$ is the attenuation rate of laser energy per unit time of the OC. The so-called “soft start” of an RF signal can increase the time of $\delta_{0}$ stabilization at the minimum value in the resonator, so as to suppress the abnormal output of the laser.

## 3. Experimental Process and Results

### 3.1. Experimental Facility

**Figure 3:**
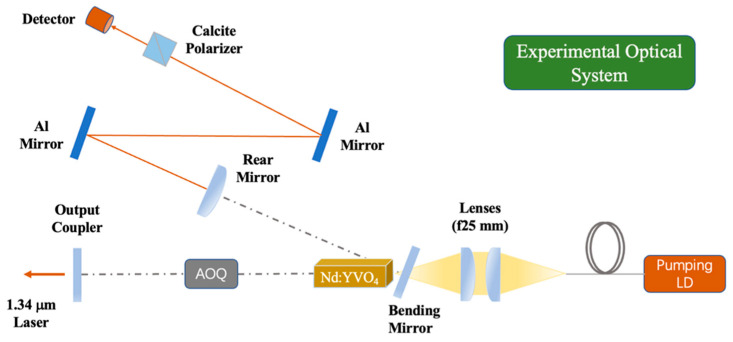
Schematic illustration of the experimental setup.

In this experiment, an LD end-pumped 1342 nm acousto-optic Q (AOQ)-switched [9] Nd:YVO_4_ laser was constructed, as shown in Figure 3.

The beam propagation factor M^2^ of the LD pump source is 7.76, and its operating temperature is about 21 °C. The coupling system consists of two focal lenses. The diameter and length of Nd:YVO_4_ are 3 mm and 6 mm with a doping concentration of 0.6 at%, respectively. Note that there is no undoped part attached at the end of the Nd:YVO_4_ crystal, and the end surface of the Nd:YVO_4_ crystal has a 2° inclination. The diameter of the pump spot is about 850 μm [10].

The cavity is designed as a V-shaped folded configuration composed of three mirrors. Compared with the F-P cavity and the flat–concave cavity, the cavity has the advantages of low loss, strong transverse mode discrimination and high output power, and the V-shaped cavity can adapt to the thermal lens effect of the laser crystal. A V-type cavity is chosen as the resonator, which includes a plano-convex rear mirror (RM), a flat output coupler (OC) and a tilted flat mirror [11]. The total length of the oscillator is about 127 mm. The RM curvature radius and OC reflectance are 200 mm and 95%, respectively. The outputted 1342 nm pulsed laser is reflected through two aluminum mirrors and then passes through a calcite polarizer. The reason for not using dielectric-coated mirrors is their feature of polarized selection. The p-polarized light is received by a semiconductor-based detector. The experiment was carried out at an ambient temperature of 22 °C.

**Figure 4:**
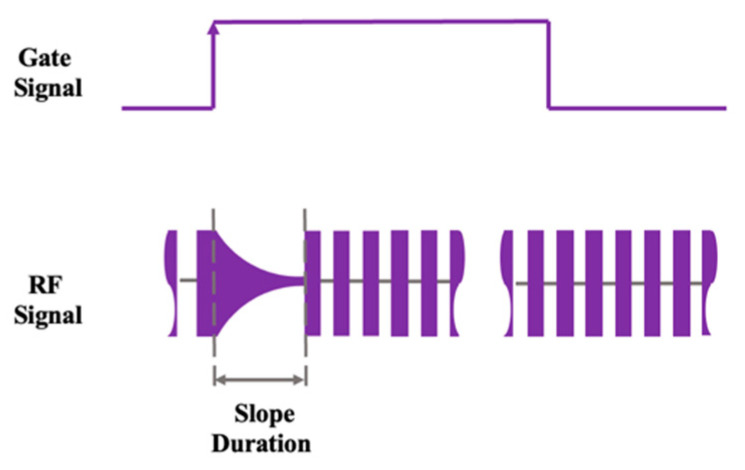
Gate signal and RF signal in our experiment.

The schematics of a gate signal and an RF signal are shown in Figure 4.

In order to obtain the high-quality practical laser output, the giant pulse component in laser emission must be suppressed by using the procedure called “Soft Starting”. As shown in the figure, the RF signal exhibits the right-oriented triangle feature. In this experiment, we tried to find whether the giant pulse of an outputted 1342 nm laser and abnormal horizontal polarization emission can be suppressed or not by adjusting the slope duration of an RF signal [12]. Note that the slope duration of the RF signal was adjusted by the control unit in a Q-switch driver.

### 3.2. Experimental Results

The laser output was realized by using the experimental optical system. The authors found that there was an abnormal horizontally polarized light output and giant pulse output before the expected vertical polarized light output after the gate was opened. The traditional method of eliminating horizontally polarized light is to add a vertical polarizer in the laser optical route to filter out the abnormal horizontally polarized light. In this paper, the authors proposed an approach to suppress the output of horizontally polarized light and giant pulse by adjusting the slope duration of the RF signal. Compared with the method of adding a polarizer, no elements are added in the optical path in this study, ensuring that the laser system is relatively simple, and the quality of the normal output laser is not affected. The inhibitory effects of the slope duration of RF signals on the generation of giant pulses [13] and lasing emission along horizontal polarization directions will be introduced in the following sub-sections. The oscillation time measurement error of horizontally polarized light is about 50 μs. The setup error of the RF signal varies with the range. When the measured slope duration value is in the order of microseconds, the setting accuracy is about 0.5 μs.

#### 3.2.1. Suppression of Giant Pulses in the Vertical Polarization Direction

**Figure 5:**
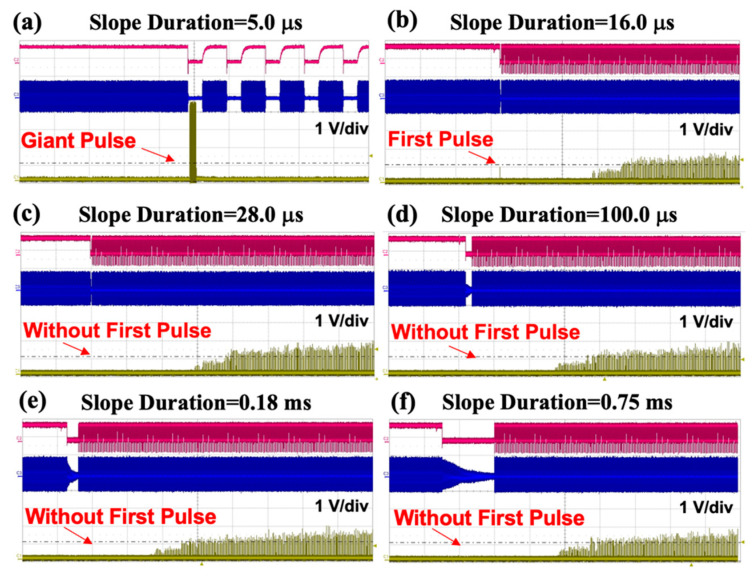
(**a**) When slope duration = 5.0 μs, a vertical polarizer is added to the output end of the laser. (**b**) Slope duration adjusted to 16.0 μs. (**c**) Slope duration adjusted to 28.0 μs. (**d**) Slope duration adjusted to 100.0 μs. (**e**) Slope duration adjusted to 0.18 ms. (**f**) Slope duration adjusted to 0.75 ms (red line: gate signal; blue line: RF signal; yellow line: vertically polarized light).

**Figure 6:**
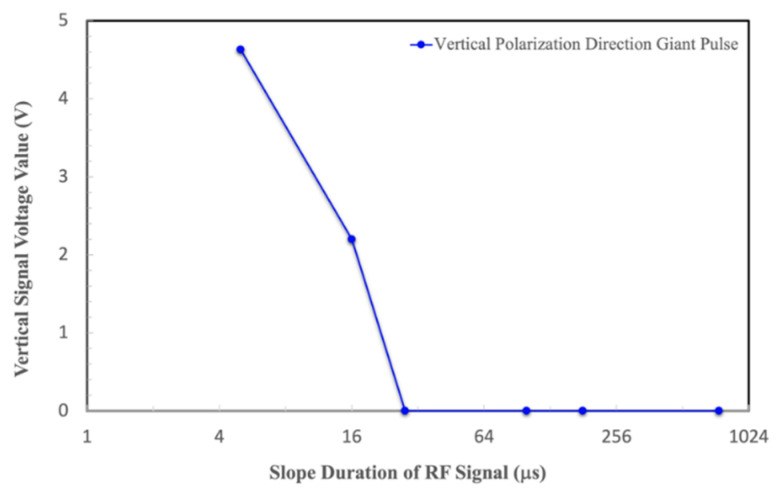
Slope duration of RF signals versus the giant pulse intensity along the vertical polarized direction.

A vertical polarizer was added into the optical route to explore the relationship between the slope duration of RF signals and the generation of giant pulses [14,15,16]. As shown in Figure 5 and Figure 6, when the slope duration is adjusted to as small as 5 μs, a serious “giant pulse” can be observed along the vertical polarization direction when the RF gate signal begins to work.

When the slope duration was stretched to 16 μs, the giant pulse in the vertical polarization direction disappeared, but an obvious vertically polarized “first pulse” can still be seen. When the slope duration was increased to be larger than 28 μs, even a small first pulse along the vertical polarization direction could not be found anymore. The results show that the giant pulse in the vertical polarization direction can be effectively suppressed by adopting the suitable RF duration.

#### 3.2.2. Suppression of Abnormal Output Light in the Horizontal Polarization Direction

Under normal conditions, the laser system outputs vertically polarized light after the gate signal was opened. However, the authors found that the abnormal horizontally polarized light and giant pulse were outputted before the expected vertical polarized light output after the gate was opened. Therefore, such an abnormal output horizontally polarized emission and horizontal giant pulse need to be eliminated by adjusting the slope duration of the RF signal.

**Figure 7:**
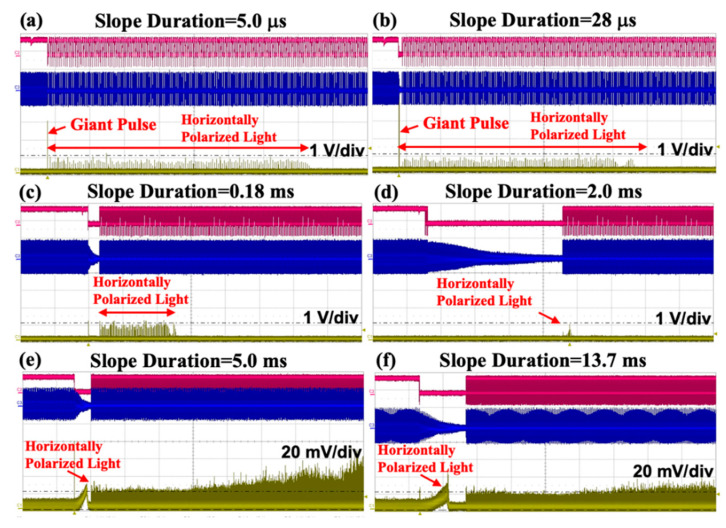
(**a**) When slope duration = 5.0 μs, a horizontal polarizer is added to the output end of the laser. (**b**) Slope duration adjusted to 28.0 μs. (**c**) Slope duration adjusted to 0.18 ms. (**d**) Slope duration adjusted to 2.0 ms. (**e**) Slope duration adjusted to 5.0 ms. (**f**) Slope duration adjusted to 13.7 ms. (**e**,**f**) are 50 times magnification (red line: gate signal; blue line: RF signal; yellow line: horizontally polarized light).

A horizontal polarizer was added into the experimental device to explore the relationship between the slope duration of RF signals and the abnormal horizontally polarized light, as well as the giant pulse. The experimental results are shown in Figure 7. With an increase in the slope duration [17], the giant pulse in the horizontal direction decreases dramatically. The giant pulse in the horizontal polarization direction almost disappears when the slope duration is greater than 0.18 ms.

As can be seen from Table 1, when the slope duration of the initial RF signal is 0 ms, the horizontally polarized light voltage value of the abnormal output is 4.557 V. With the increasing slope duration of the RF signal, the horizontally polarized light voltage value of the abnormal output gradually decreases. When the slope duration of the RF signal is 13.7 ms, the horizontally polarized light voltage value of the abnormal output is reduced by two orders of magnitude compared with the initial state. Note that the voltage value represents the abnormal light output intensity.

**Figure 8:**
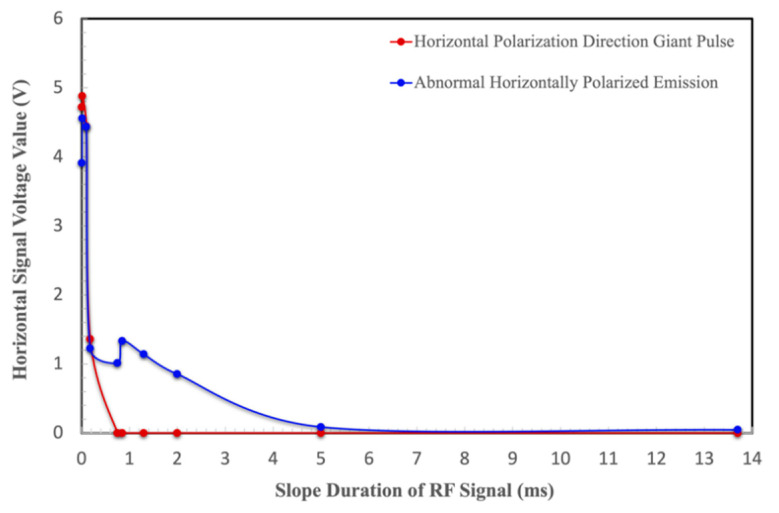
Giant pulse and abnormal output lasing emission along the horizontally polarized direction versus the slope duration of RF signals.

The experimental results show that when the slope duration gradually increases, the lasing span of the abnormal output horizontally polarized light decreases. When the slope duration was adjusted from 5 to 28 μs, the abnormal output horizontally polarized light was kept alive; when the slope duration was greater than 2 ms, the abnormal output horizontally polarized light decreased to a very small level; when the slope duration was equal to 5 ms, the abnormal output horizontally polarized light became almost zero. When the slope duration was greater than 13.7 ms, the abnormal output horizontally polarized light can be effectively suppressed, and the experimental results are shown in Figure 7 and Figure 8.

## 4. Analyses and Discussions

By carrying out the theoretical analyses and verification, we have found the cause of the abnormal output horizontally polarized emission. In this study, the wavelength of the pump light matches the absorption peak of the working medium well, so that the absorbed power of the pump diode lasers is somewhat large [18]. About 20% of the energy is thought to be deposited in the Nd:YVO_4_ crystal, which forms an uneven distribution of heat in the Nd:YVO_4_ crystal [19]. Generally, the heat dissipation of the crystal surface is fast, but the heat dissipation effect inside the crystal is poor. An obvious temperature gradient forms in the crystal cross-section, which leads to the variation of the refractive index of the Nd:YVO_4_ crystal [20], resulting in the thermally induced lens effect of the crystal. Such an effect of the crystal can affect the laser conversion efficiency, cavity stability and laser beam quality.

**Figure 9:**
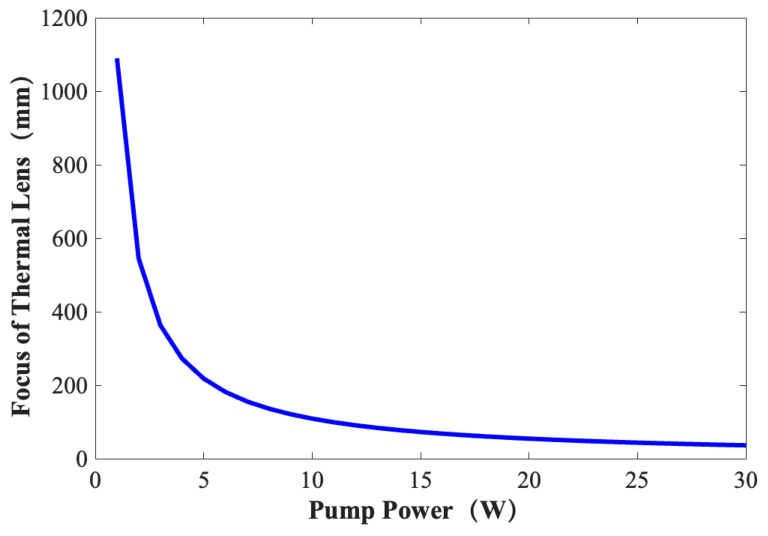
Relationship between focal length of thermal lens and average pump power.

According to the theoretical model for describing the solid-state laser thermally induced lens proposed in relevant studies [21], it can be seen that under the action of pumping light, the working solid-state medium is equivalent to a thermal lens with the focal length of *F*. *F* changes with the pump light power *P*, and the following formula is expressed to calculate the focal length of a thermal lens:

(6) $$F=\frac{\pi k_{c}\omega_{p}^{2}}{P\eta (dn/dT)\left[1-exp(-\alpha_{p}l)\right]}$$

where $k_{c}$ is the thermal conductivity, $\omega_{p}$ is the optical waist, dn/dT is the refractive index change rate with temperature, $\alpha_{p}$ is the absorption coefficient of the pump light, l is the length of the Nd:YVO_4_ crystal, P is the pump power and η is the conversion coefficient of the pump power into heat. According to Formula (6), the parameter values in the thermal lens formula are shown in Table 2, and the relationship between the focal length of a thermal lens in the cavity and the pump power can be obtained as shown in Figure 9.

In Figure 9, it can be seen that the thermal lens effect is weak and the focal length of a thermal lens is large when the pump power is relatively small. With the increase in the pump power, the thermal effect is enhanced, and the focal length of the thermal lens is rapidly reduced. When the high power reaches more than 10 W [22], the focal length of a thermal lens changes relatively slowly with the pump power. According to the current research on the output characteristics of a high-power pumped Nd:YVO_4_ solid-state laser, compared with a high low-power pumped Nd:YVO_4_ solid-state laser, we found that the thermal lens effect [23] of our high-power pumped Nd:YVO_4_ solid-state laser has a greater thermal influence on the laser output.

In order to facilitate the analyses, the temperature distribution in the laser medium can be simplified into a one-dimensional temperature distribution along the radial direction on the cross-section, which can be expressed by the following:

(7) $$T_{\left(x,y\right)}=T_{W}+\frac{\eta P_{in}}{4\pi Kl}[1-(x^{2}+y^{2})]$$

In Formula (7), l is the length of the laser medium, $P_{in}$ is the power dissipated by the medium, η is the heat consumption power coefficient, $T_{W}$ is the surface temperature of the medium, K is the thermal conductivity and x,y are the cartesian coordinates normalized to the radius in the cross-section of the medium.

The main parameters characterizing the thermal lens effect include the focal length f of the thermal lens and the focal power D, which is defined as

(8) $$D=\frac{1}{f}\approx {\left.-l\left[a\left(n_{0}-1\right)+\frac{dn}{dT}+\varepsilon_{r,\varphi }\right]\frac{d^{2}T}{dr^{2}}\right|}_{r=0}$$

In Formula (8), a is the linear expansion coefficient, $\frac{dn}{dT}$ is the temperature coefficient of the refractive index, $n_{0}$ is the normal temperature refractive index of the laser crystal, $\varepsilon_{r,\varphi }$ is related to thermal stress birefringence, r is the radial distance and ${\left.-la(n_{0}-1)\frac{d^{2}T}{dr^{2}}\right|}_{r=0}$ stands for face bending due to heat.

By solving the above formulas, the relationship between crystal temperature and the equivalent focal length of the thermal lens can be further obtained. It is estimated that when the pump energy is large, the heat accumulation in the crystal will cause the equivalent focal length of the thermal lens generated by the crystal to become very small, which will affect the oscillation output of the laser.

The existence of the thermal lens effect seriously influences the stability of a resonator and output characteristics of the laser because of the thermally induced lens. When the thermal lens effect of the working medium becomes serious, the resonator will be in the unstable region, and the laser oscillation might not be formed in the oscillator. According to the above explanation, it is better explained that the thermal lens effect of the working medium is intense when the slope duration of the RF signal is relatively small. At this time, the resonator is in an unstable region and cannot produce the expected vertical polarized light output. As the slope duration of the RF signal increases, the thermal lens effect of the working medium decreases. The resonator is in a stable region, thus producing the desired vertical polarized light output.

In this study, a ray transfer matrix (ABCD matrix) methodology [24] has been applied to investigate the relationship between the radius of a fundamental beam at the RM and the focal length of a thermally induced lens of our Nd:YVO_4_ crystal [25].

(9) $$\left[\begin{array}{cc} A & B\\ C & D \end{array}\right]=\left[\begin{array}{cc} A_{n} & B_{n}\\ C_{n} & D_{n} \end{array}\right]\ldots \left[\begin{array}{cc} A_{2} & B_{2}\\ C_{2} & D_{2} \end{array}\right]\left[\begin{array}{cc} A_{1} & B_{1}\\ C_{1} & D_{1} \end{array}\right]$$

(10) $$\left[\begin{array}{cc} A & B\\ C & D \end{array}\right]=\left[\begin{array}{cc} 1 & d_{1}-z\\ 0 & 1 \end{array}\right]\left[\begin{array}{cc} 1 & 0\\ \frac{2}{R_{1}} & 1 \end{array}\right]\left[\begin{array}{cc} 1 & d_{2}\\ 0 & 1 \end{array}\right]\left[\begin{array}{cc} 1 & \frac{d_{3}}{n_{0}}\\ 0 & 1 \end{array}\right]\left[\begin{array}{cc} 1 & 0\\ -\frac{1}{f_{T}} & 1 \end{array}\right]\left[\begin{array}{cc} 1 & \frac{L-d_{3}}{n_{0}}\\ 0 & 1 \end{array}\right]\;\left[\begin{array}{cc} 1 & d_{4}\\ 0 & 1 \end{array}\right]\left[\begin{array}{cc} 1 & \frac{L_{A}}{n_{A}}\\ 0 & 1 \end{array}\right]\left[\begin{array}{cc} 1 & d_{5}\\ 0 & 1 \end{array}\right]\left[\begin{array}{cc} 1 & 0\\ \frac{2}{R_{2}} & 1 \end{array}\right]\left[\begin{array}{cc} 1 & d_{5}\\ 0 & 1 \end{array}\right]\left[\begin{array}{cc} 1 & \frac{L_{A}}{n_{A}}\\ 0 & 1 \end{array}\right]\left[\begin{array}{cc} 1 & d_{4}\\ 0 & 1 \end{array}\right]\left[\begin{array}{cc} 1 & \frac{L-d_{3}}{n_{0}}\\ 0 & 1 \end{array}\right]$$

(11) $$\omega_{f_{T}}=\left\{\begin{array}{c} {\left(\frac{\lambda }{\pi }\right)}^{0.5}\frac{\sqrt{\left|B\right|}}{{\left[1-{\left(\frac{A+D}{2}\right)}^{2}\right]}^{0.25}},\;\;\;if\left|\frac{A+D}{2}\right|<1\\ 0,\;\;\;others \end{array}\right.$$

**Figure 10:**
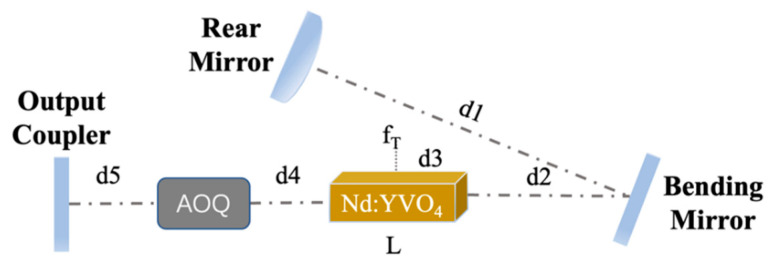
Schematic diagram of Nd:YVO_4_ laser resonator.

The parameters in Figure 10 and Equations (9)–(11) are explained as follows: $d_{1}$ is the distance between the RM and the bending mirror; $d_{2}$ is the distance between the bending mirror and the Nd:YVO_4_ crystal; $d_{3}$ is the distance between the focal point of pump light and the right end of the Nd:YVO_4_ crystal; $f_{T}$ is the focal length of the thermally induced lens; L is the length of the Nd:YVO_4_ crystal; $d_{4}$ is the distance between the Nd:YVO_4_ crystal and the AOQ; $d_{5}$ is the distance between the AOQ and the OC; $R_{1}$ is the radius of curvature of the RM; $R_{2}$ is the radius of curvature of the OC; $L_{A}$ is the length of the AOQ; $n_{A}$ is the refractive index of AOQ; $n_{0}$ is the refractive index of air; $r_{1}$ is the radius of curvature of the bending mirror; $\omega_{f_{T}}$ is the radius of a fundamental beam at RM.

According to the above formula, the author inputs the parameters of the resonator system into the simulation software (Mathcad 2010) and obtains the relationship between the radius of a fundamental beam at RM and the focal length of the thermal lens.

**Figure 11:**
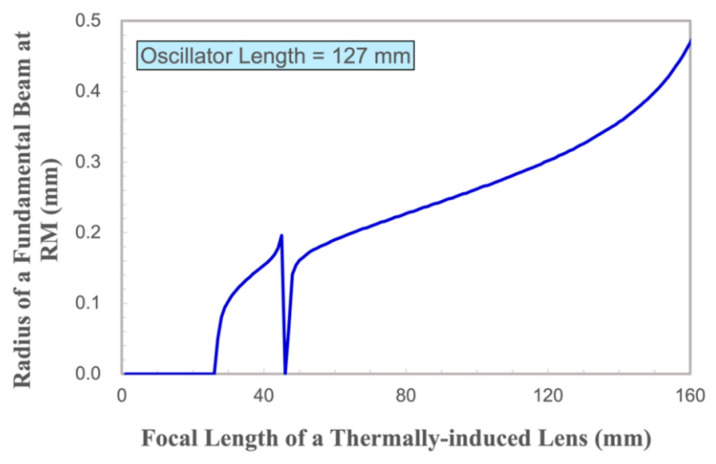
Radius of a fundamental beam at the RM versus the focal length of a Thermally Induced Lens of the Nd:YVO_4_ crystal in our study.

The verification results are shown in Figure 11. When the focal length of a thermally induced lens is less than 26 mm, the laser oscillation cannot be formed in the resonator. It is proved that, since the excited cross-section area of the Nd:YVO_4_ crystal in the direction of the c-axis is about ten times that of the a-axis, the thermally induced lens effect in the vertical direction is much more severe than that in the horizontal direction, and the focal length of a thermally induced lens is thought to be less than 26 mm for the vertical polarization. Therefore, there is no vertical polarized light output in the initial operation of the laser just after the gate signal is opened. As the thermally induced lens effect in the horizontal polarization direction is weak, the focal length of a thermally induced lens should be much greater than 26 mm in the horizontal direction. Therefore, the abnormal horizontally polarized light will be produced when the gate signals from the artificial control start working. Until the temperature gradient in the working medium decreases and the thermally induced lens effect weakens, the vertical polarized emission will start and win the competition with the abnormal horizontally polarized light mode afterwards. After the vertical polarized oscillation is formed, the abnormal output horizontally polarized light can be effectively suppressed. Such theoretical analyses and verification can be used to explain the reason for abnormal horizontally polarized light when the gate signals are working.

## 5. Conclusions

**Figure 12:**
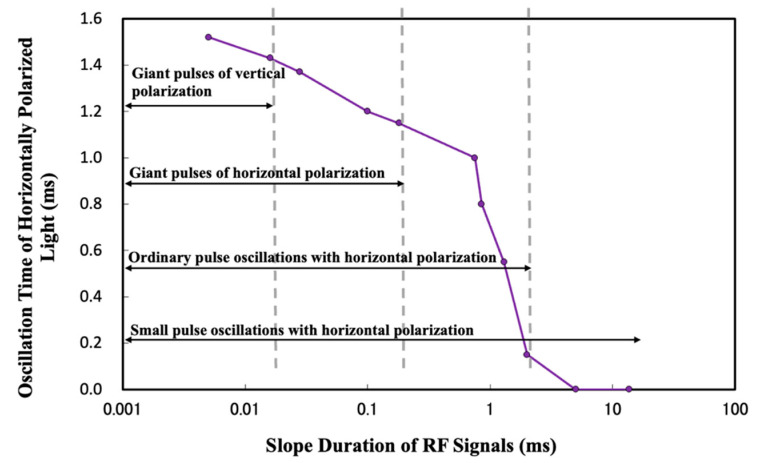
Relationship between the slope duration of the RF signal and the generation of the giant pulse and horizontally polarized emission.

After sorting out the experimental data, we find that with the increase in the slope duration of RF signals, the oscillation time of abnormal output horizontally polarized light is gradually shortened. In this process, when the slope duration is equal to 16 μs, the giant pulse along the vertical direction disappears first. When the slope duration is equal to 0.18 ms, the giant pulse polarization along the horizontal direction is effectively suppressed. When the slope duration continues to increase to 5 ms, the abnormal horizontally polarized light output becomes very weak. When the slope duration is greater than 13.7 ms, the abnormal output horizontally polarized light can be effectively suppressed [26], and the results are diagramed in Figure 12.

In this study, we find the appearance of not only a giant pulse in both the vertical and the horizontal directions when the gate signal is artificially started, but also some abnormal horizontally polarized emission outputs with a short duration caused by the thermally induced lens effect. Through the theoretical analyses and corresponding experimental verification, we carefully investigated the relationship between the slope duration of an RF signal and the generation of the horizontally polarized light, as well as the giant pulses. With the increase in the slope duration, the giant pulse gradually disappears and the duration of horizontally polarized emission gradually decreases. When the slope duration is greater than 0.18 ms, both the horizontal giant pulse and the vertical giant pulse will disappear. When the slope duration is greater than 13.7 ms, the abnormal output horizontally polarized emission can be effectively suppressed. After the horizontally polarized light is suppressed, the laser can obtain stable vertical polarized light output. The thermal lensing effect is slowed down by increasing the slope duration of the RF signal. Before the threshold slope duration, the desired vertical polarized light oscillation can be formed in the resonator, thus suppressing the output of abnormal light. The excited cross-section area of the Nd:YVO_4_ crystal in the direction of the c-axis is about ten times that of the a-axis. The thermally induced lens effect in the vertical direction is much more severe than that in the horizontal direction. Therefore, when the slope duration of the RF signal is adjusted to reduce the thermal effect, the vertical polarization direction giant pulse will disappear before the horizontal polarization direction giant pulse and horizontal polarization light.

By using a traditional method, abnormal horizontally polarized light can be eliminated by adding some polarizers to the optical system. However, such a traditional method will increase the complexity of the whole optical system. Compared with the traditional solution procedure, the effective approach proposed by the authors consists of just adjusting the slope duration of RF signals to suppress both the abnormal horizontally polarized light and the giant pulses. In such a scheme, it is unnecessary to increase any optical elements. The optical system can be constructed in a small volume, and the power and other performances of the outputted vertically polarized light will not be affected anymore. The results introduced in this paper are thought to be beneficial to improve the accuracy of laser processing.

There are still some limitations in this study. For example, the method requires a large slope duration to suppress the output of abnormal light when the pump power is larger, but this will prolong the opening time of the laser. Therefore, a better cooling scheme should be designed to accommodate the use of this method to further inhibit the thermal lens effect of the crystal and ensure the stability of the laser.

More laser crystals will produce heat deposition under the irradiation of the pump light and produce a thermal lens effect, which will seriously affect the output performance of Q-switched lasers. The above research proves the effectiveness of the method in the experiment. In the future, the authors will further theoretically investigate the relationship between the temperature gradient inside the crystal and the pump energy at the theoretical level, and then conduct theoretical analyses of the strength of a thermally induced lens. In the future, the accurate correspondence between the thermal lensing effect of laser crystal and the RF signal slope duration will be obtained, and the calculation system will be extended to increase Q-switched laser output optimization.

## Figures and Tables

**Table 1:** Relationship between the slope duration of the RF signal and the voltage value of abnormal horizontal polarization.

Slope Duration of RF Signal (ms)	0	0.1	0.85	1.3	2.0	5.0	13.7
Abnormal horizontally polarized emission voltage value (V)	4.557	4.436	1.332	1.140	0.852	0.087	0.046

**Table 2:** Parameter values in the thermal lens formula.

$k_{c}$	$\omega_{p}$	dn/dT	$\alpha_{p}$	l	η
5.4 × 10^−3^ (W/cm·K)	300 × 10^−3^ (mm)	4.67 × 10^−6^ (1/K)	1.48 (1/mm)	10 (mm)	0.3

## Data Availability

The authors confirm that the data supporting the findings of this study are available within the article.
